# Priming with *FLO8-*deficient *Candida albicans* induces Th1-biased protective immunity against lethal polymicrobial sepsis

**DOI:** 10.1038/s41423-020-00576-6

**Published:** 2020-11-05

**Authors:** Quan-Zhen Lv, De-Dong Li, Hua Han, Yi-Heng Yang, Jie-Lin Duan, Hui-Hui Ma, Yao Yu, Jiang-Ye Chen, Yuan-Ying Jiang, Xin-Ming Jia

**Affiliations:** 1 Clinical Medicine Scientific and Technical Innovation Center, Shanghai Tenth People’s Hospital, Tongji University School of Medicine, Shanghai, 200092 China; 2 School of Pharmacy, Second Military Medical University, Shanghai, 200433 China; 3 State Key Laboratory of Molecular Biology, Shanghai Institutes for Biological Sciences, Chinese Academy of Sciences, Shanghai, 200031 China

**Keywords:** Fungi infection, *Candida albicans*, Protective immunity, Thymus atrophy, Apoptosis, Fungal infection, Live attenuated vaccines

## Abstract

The morphological switch between yeast and hyphae of *Candida albicans* is essential for its interaction with the host defense system. However, the lack of understanding of host–pathogen interactions during *C. albicans* infection greatly hampers the development of effective immunotherapies. Here, we found that priming with the *C. albicans FLO8-*deficient (*flo8*) mutant, locked in yeast form, protected mice from subsequent lethal *C. albicans* infection. Deficiency of Dectin-2, a fungus-derived α-mannan recognition receptor, completely blocked *flo8* mutant-induced protection. Mechanistically, the *flo8* mutant-induced Dectin-2/CARD9-mediated IL-10 production in DCs and macrophages to block thymus atrophy by inhibiting the *C. albicans*-induced apoptosis of thymic T cells, which facilitated the continuous output of naive T cells from the thymus to the spleen. Continuous recruitment of naive T cells to the spleen enhanced Th1-biased antifungal immune responses. Consequently, depletion of CD4+ T cells or blockade of IL-10 receptor function using specific antibodies in mice completely blocked the protective effects of *flo8* mutant priming against *C. albicans* infection. Moreover, mannans exposed on the surface of the *flo8* mutant were responsible for eliciting protective immunity by inhibiting the *C. albicans*-induced apoptosis of thymic T cells to sustain the number of naive T cells in the spleen. Importantly, priming with the *flo8* mutant extensively protected mice from polymicrobial infection caused by cecal ligation and puncture (CLP) by enhancing Th1-biased immune responses. Together, our findings imply that targeting *FLO8* in *C. albicans* elicits protective immune responses against polymicrobial infections and that mannans extracted from the *flo8* mutant are potential immunotherapeutic candidate(s) for controlling infectious diseases.

## Introduction

Sepsis is the systemic inflammatory response syndrome caused by bacterial, viral, or fungal infections.^1^ Although hundreds of clinical trials have been conducted, no effective new therapies against sepsis have been approved. Invasive fungal infections, especially candidemia, have become a major cause of morbidity and mortality in the past few decades, and these fungi are difficult to target pharmacologically. Accumulating evidence supports that effective therapies against invasive fungal infections require the development of immunotherapeutic strategies, which could be combined with antifungal chemotherapy.^2,3^ Recent studies have shown that vaccination with live attenuated vaccines such as Bacillus Calmette-Guérin, the measles vaccine, the oral polio vaccine, or commensal intestinal fungi such as *Candida albicans* and *Saccharomyces cerevisiae* can protect the host from infections caused by various species of microorganisms,^4–6^ which makes it possible to develop immunopotentiators or vaccines against polymicrobial sepsis from attenuated pathogens or human commensal microorganisms. For *C. albicans*, a leading commensal fungal pathogen affecting humans, the transition between yeast and hyphal forms is critical for its pathogenicity in life-threatening invasive candidiasis, and this disease has a high mortality rate, exceeding 40%.^7–9^ Current studies show that *C. albicans* yeast cells are suitable for early colonization and dissemination, while hyphal forms are required for tissue penetration and organ-seated infection. Therefore, most *C. albicans* mutants locked in either morphological state are less virulent in mouse bloodstream infection models.^10^ Inhibiting fungal morphological transformation is an important means of inhibiting fungal virulence and immune escape. However, despite intense research efforts, the development of efficient antifungal immunotherapies has fallen behind in part due to an insufficient understanding of the host–pathogen interactions and the mechanisms underlying immune escape and the induction of protective immunity during fungal infections.

The transcription factor Flo8p is known to be essential for hyphal development in *C. albicans*, and the *flo8* null mutant kept in yeast form is hypovirulent in the bloodstream infection mouse model.^11^ However, we found that the *flo8* null (*flo8*) mutant was fully virulent in a systemic candidiasis mouse model deficient for caspase recruitment domain family member 9 (CARD9), which is well known to operate downstream of several immunoreceptor tyrosine-based activation motif-associated C-type lectin receptors (CLRs), including Dectin-1, Dectin-2, and Dectin-3, to mediate antifungal immune responses in mice and humans.^12–16^ Therefore, we hypothesize that the *flo8* mutant shapes host immune responses toward a host-protective type. In this case, the *flo8* mutant may elicit immune responses to protect hosts from subsequent lethal infection. Our findings suggested that the *flo8* mutant is an effective immunopotentiator for controlling polymicrobial sepsis and that mannans extracted from this mutant might provide potential immunotherapeutic candidate(s) for controlling infectious diseases.

## Results

### Priming with the *flo8* mutant protects mice from *C. albicans* infection through Dectin-2

**Fig. 1:**
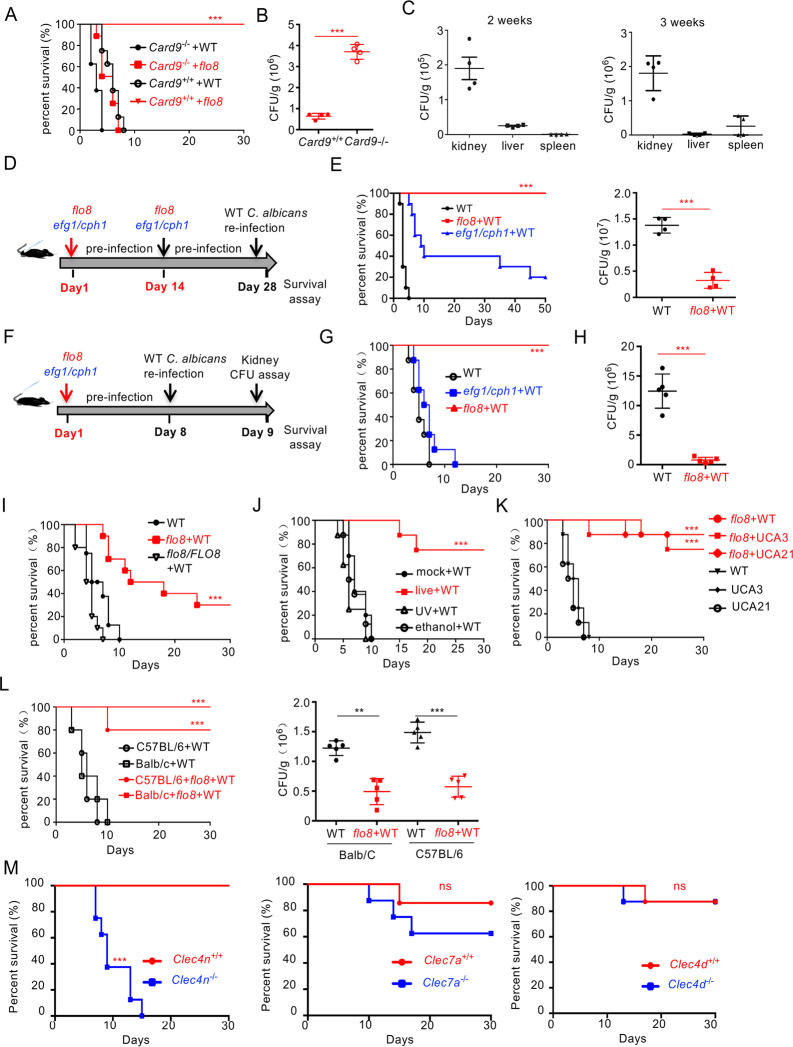
Live nonhyphal *flo8* null mutant primary challenge protects mice from candidiasis depending on Dectin-2. **A** Survival curves of wild-type (*Card9*^+/+^) and *Card9*-deficient (*Card9*^−/−^) mice, which were intravenously infected with 5 × 10^5^ CFUs of *flo8* mutant (*n* = 8). **B** Kidney fungal burden of *Card9*^−/^^−^ mice infected with 5 × 10^5^ CFUs of *flo8* for 24 h (*n* = 4). **C** Kidney fungal burden of mice infected with 5 × 10^5^ CFUs of *flo8* for 2 or 3 weeks (*n* = 4). **D**, **E** Strategy for the primary infection and reinfection. Mice were intravenously infected with live 5 × 10^5^ CFUs of the *flo8* or *efg1/cph1* mutant twice at an interval of 14 days and reinfected with 5 × 10^5^ CFUs of WT *C. albicans* (SC5314) at day 28 (**D**). The kidney fungal burden (*n* = 4, right) was determined at day 30, and the survival rate (*n* = 10, left) was evaluated for another 50 days (**E**). **F**–**H** Strategy for primary infection and reinfection. Mice were intravenously infected with 5 × 10^5^ CFUs of the live *flo8* or *efg1/cph1* mutant for 7 days and then reinfected with 5 × 10^5^ CFUs of lethal WT *C. albicans* at day 8 (**F**). The survival rate (**G**) and renal fungal burden (**H**) of mice were determined (*n* = 8). **I** Survival curves of C57BL/6 mice that were previously inoculated with the *flo8* mutant or *FLO8* revertant strain *flo8/FLO8* (1 × 10^5^ CFUs) and reinfected with 5 × 10^5^ CFUs of WT *C. albicans* (*n* = 8). **J** Survival curves of C57BL/6 mice, which were primary challenged with the live, UV-inactivated or 75% ethanol-inactivated *flo8* (5 × 10^5^ CFUs) mutant and then rechallenged with 5 × 10^5^ CFUs of WT *C. albicans* (*n* = 8). **K** Survival curves of mice that were previously infected as described in (**F**) and reinfected with 5 × 10^5^ CFUs of WT *C. albicans* or the clinical isolate UCA3 or UCA21 (*n* = 8). **L** Survival curves (*n* = 10) and kidney fungal burdens of C57BL/6 and Balb/C mice, which were primarily infected and reinfected as described in (**F**). **M** Survival curves of *Clec4n*^−/−^, *Clec7a*^−/−^ and *Clec4e*^−/−^ mice, which were infected with 5 × 10^5^ CFUs of *flo8* for 7 days and reinfected with 5 × 10^5^ CFUs of WT *C. albicans* (*n* = 8). Bars, mean ± SEM. *p < 0.05, **p < 0.01, ***p < 0.001, ns not significant, determined by the log rank (Mantel–Cox) test (**A**, **E**, **G**, **I**, **J**, **K**, **L**, **M**) or the unpaired *t* test (**B**, **E**, **H**, **L**). Similar results were obtained in at least two independent experiments

Consistent with previous studies,^11,17^ disruption of the *FLO8* gene in *C. albicans* completely blocked the serum-induced yeast-to-hyphae transition at 37 °C (Fig. S1A). Furthermore, infection with the *flo8* mutant did not result in any fatality in wild-type mice for up to 30 days (Fig. 1A). However, infected mice failed to completely clear the *flo8* mutant in the kidney, liver, and spleen for up to 21 days (Fig. 1C), suggesting that the *flo8* mutant is a hypovirulent strain. However, we surprisingly observed that the *flo8* mutant had strong pathogenicity in CARD9-deficient mice (Fig. 1A, B). These data suggest that the attenuated virulence of the *flo8* mutant in wild-type mice may have been due to the activation of CARD9-mediated protective immune responses. These protective benefits indicated that priming with the *flo8* mutant may protect the host against a subsequent lethal infection.

To examine the above hypothesis, we employed the priming strategy with two inoculations spaced at an interval of 14 days, as shown in Fig. 1D. All mice primed with the *flo8* mutant (5 × 10^5^ CFUs) survived for more than 30 days after infection with the fully virulent *C. albicans* strain SC5314 (Fig. 1E). Consistently, priming with the *flo8* mutant significantly reduced the kidney fungal load in mice after reinfection with *C. albicans* (Fig. 1E). However, priming with the *efg1/cph1* mutant, which was also locked in yeast form,^18,19^ slightly increased the survival of infected mice (Fig. 1E). When the interval of inoculation was decreased to 7 days (Fig. 1F), sufficient induction of immune protection by the *flo8* mutant was observed (Fig. 1G, H). In contrast, priming with the *efg1/cph1* mutant had no protection against *C. albicans* reinfection (Fig. 1G). Moreover, when the interval of inoculation was decreased to 3 days, priming with the *flo8* mutant had no protection against *C. albicans* reinfection (Fig. S1B).

To determine whether the protection of the *C. albicans* mutant was controlled by Flo8p, mice were primarily challenged with the *flo8* mutant or the *FLO8* revertant strain (1 × 10^5^ CFUs, Fig. 1I). Priming with a low dose of the *flo8* mutant was partially protective (Fig. 1I). However, the *FLO8* revertant strain had no protection against *C. albicans* reinfection (Fig. 1I). In addition, the *flo8* mutant inactivated by UV or ethanol had no protection (Fig. 1J). Together, these data suggest that the live *flo8* mutant provided dose-dependent protection against secondary *C. albicans* infection and that this protection was specifically regulated by Flo8p.

We then examined whether the immunoprotection induced by the *flo8* mutant was dependent on subsequent challenge with specific *C. albicans* strains or mouse species. We observed that the *flo8* mutant had comparable protective influences on the survival rate after reinfection with clinical *C. albicans* isolate UCA3 or UCA21 (Fig. 1K). Comparable protective benefits induced by the *flo8* mutant were also observed in BALB/C and C57BL/6 mice (Fig. 1L). Collectively, these data indicated that priming with the *flo8* mutant could protect the host against a subsequent lethal infection, and this protection was independent of the strain or mouse species utilized.

To explore whether the immunoprotection induced by the *flo8* mutant against *C. albicans* infection was mediated by CLR signaling, we utilized mice deficient for Dectin-1, Dectin-2, or Dectin-3. We found that Dectin-2 deficiency completely blocked *flo8* mutant-induced protection, resulting in a significantly lower survival rate of mice after *C. albicans* infection than that of control mice (Fig. 1M). However, deficiency of Dectin-1 or Dectin-3 had no significant influence on *flo8* mutant-induced protection (Fig. 1M). Together, these data implied that priming with the *flo8* mutant protected mice from *C. albicans* infection through Dectin-2.

### Priming with the *flo8* mutant blocks *C. albicans*-induced thymus atrophy by inhibiting T-cell apoptosis

**Fig. 2:**
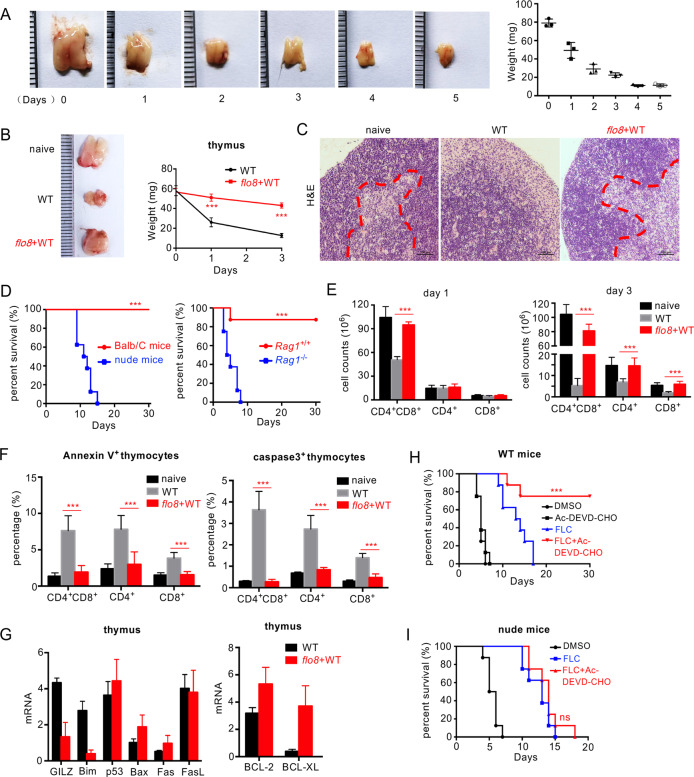
Thymus atrophy abrogation by prior inoculation with the *flo8* mutant is required for resistance to candidiasis. **A** Photograph of a representative thymus and thymic weights of mice infected with 5 × 10^5^ CFUs of WT *C. albicans* for 0–5 days. **B** Photograph of a representative thymus and thymic weights of mice that were primarily challenged with 5 × 10^5^ CFUs of *flo8* and then rechallenged with 5 × 10^5^ CFUs of WT *C. albicans* for 24 h. **C** Histological analysis (H&E staining) of thymuses from mice treated as described in (**B**). The dotted red line indicates the division between the cortex and medulla. **D** Survival curves of nude mice (*n* = 10) and *Rag1*^−/−^ mice (*n* = 8) infected with 5 × 10^5^ CFUs of the *flo8* mutant for 7 days and reinfected with 5 × 10^5^ CFUs of WT *C. albicans*. **E** FACS quantification of T cells stained with anti-CD4 and anti-CD8 antibodies in the thymuses of mice infected with 5 × 10^5^ CFUs of the *flo8* mutant for 7 days and reinfected with 5 × 10^5^ CFUs of WT *C. albicans* for 1 or 3 days (*n* = 5). **F** FACS analysis of Annexin V^+^ cells or Caspase-3^+^ apoptotic T cells in thymus organs from mice infected with the 5 × 10^5^ CFUs of the *flo8* mutant for 7 days and reinfected with 5 × 10^5^ CFUs of WT *C. albicans* for 3 days (*n* = 5). **G** RT-PCR analysis of the expression of proapoptotic genes (GILZ, Bim, p53, Bax, Fas, FasL) and antiapoptotic genes (Bcl-2, BCL-XL) in the thymuses of mice infected with the 5 × 10^5^ CFUs of the *flo8* mutant for 7 days and reinfected with 5 × 10^5^ CFUs of WT *C. albicans* for 1 day (*n* = 5). Survival curves of Balb/C (**H**) and nude mice (**I**) infected with 5 × 10^5^ CFUs of WT *C. albicans* and treated with drugs once a day for 3 days. Fluconazole (FLC), 0.5 mg/kg, Ac-DEVD-CHO, 10 mg/kg (*n* = 8). Bars, mean ± SEM. *p < 0.05, **p < 0.01, ***p < 0.001, ns not significant, determined by one-way ANOVA (**E**, **F**), the log rank (Mantel–Cox) test (**D**, **H**, **I**), or the unpaired *t* test (**B**). Similar results were obtained in at least two independent experiments

Severe thymus atrophy, largely reflecting intense lymphocyte depletion caused by massive cortical T-cell apoptosis, is commonly observed in a variety of acute infections, including viral diseases, bacterial infections, and fungal infections.^20–22^ Here, we observed a persistent and significant decrease in thymus size and weight until death in mice infected with a lethal dose of *C. albicans* (Fig. 2A, B). Histological examination of thymuses from infected mice revealed a marked disappearance of lymphocytes from the thymic cortex (Fig. 2C). These data suggest that *C. albicans* infection induced irreversible thymus atrophy in mice. However, we surprisingly found that priming with the *flo8* mutant completely prevented mice infected with *C. albicans* from thymus atrophy, as the thymuses of these mice were comparable in size, weight, and cortical lymphocyte appearance to those in uninfected mice (Fig. 2B, C). To determine whether the protective responses induced by the *flo8* mutant were dependent on the thymus, we prechallenged athymic nude mice or mice, which lack mature T and B cells, with deficiency of recombination-activating gene 1 (*RAG1*). We observed that priming with the *flo8* mutant had no protection against *C. albicans* infection in nude or *RAG1*-deficient mice (Fig. 2D). These data indicate that the thymus is required for the protective responses induced by the *flo8* mutant.

To further explore the influences of *C. albicans* infection and *flo8* mutant priming on the thymus, we investigated whether *C. albicans* infection selectively affected certain thymocyte subpopulations. Consistent with thymus atrophy, the absolute numbers of immature CD4^+^CD8^+^ T cells and mature CD4^+^ and CD8^+^ T cells were significantly decreased, especially at day 3 after *C. albicans* infection (Figs. 2E and S2A). However, priming with the *flo8* mutant successfully sustained the numbers of CD4^+^CD8^+^, CD4^+^, and CD8+ T cells in the thymuses of reinfected mice (Figs. 2E and S2A). Together, these data suggest that priming with the *flo8* mutant blocks thymus atrophy by inhibiting decreased T-cell numbers caused by *C. albicans* infection.

It is well documented that a number of bacterial, viral and parasitic infections decrease T-cell numbers through the apoptosis pathway.^22^ To determine whether apoptosis contributes to the loss of T cells, thymus organs were collected from mice to examine the expression of well-characterized apoptosis cell markers, including Annexin V, caspase-3, proapoptotic genes (GILZ, Bim, p53, Bax, Fas, and FasL), and antiapoptotic genes (Bcl2 and BCL-XL). We found that *C. albicans* infection significantly increased the frequency of Annexin V and caspase-3 expression in CD4^+^CD8^+^, CD4^+^ or CD8+ T cells (Figs. 2F and S2B, C) and selectively increased the expression levels of proapoptotic genes, including GILZ, Bim, p53, and FasL, in the thymus (Fig. 2G). In contrast, priming with the *flo8* mutant significantly decreased the frequency of Annexin V^+^ and caspase-3^+^ T cells in infected mice (Figs. 2F and S2B, C). Moreover, this priming significantly reduced the expression of the proapoptotic genes GILZ and Bim and significantly increased the expression of the antiapoptotic genes BCL-2 and BCL-XL (Fig. 2G). Thus, these data implied that priming with the *flo8* mutant blocked *C. albicans*-induced thymus atrophy by prohibiting the apoptosis of CD4^+^CD8^+^, CD4^+^, and CD8^+^ T cells.

Blockade of infection-induced T-cell apoptosis by a caspase-3 inhibitor has been reported to be highly beneficial in sepsis treatment.^23^ Consistently, we found that treatment with the caspase-3 inhibitor Ac-DEVD-CHO combined with the antifungal agent fluconazole for 3 days significantly improved the survival rate of mice infected with lethal *C. albicans* (Fig. 2H). However, this combination did not improve the survival rate of athymic nude mice (Fig. 2I). These data suggested that inhibiting T-cell apoptosis was decisive for conferring host resistance to *C. albicans* infections.

### Priming with the *flo8* mutant maintains the continuous output of naive T cells from the thymus to the spleen

**Fig. 3:**
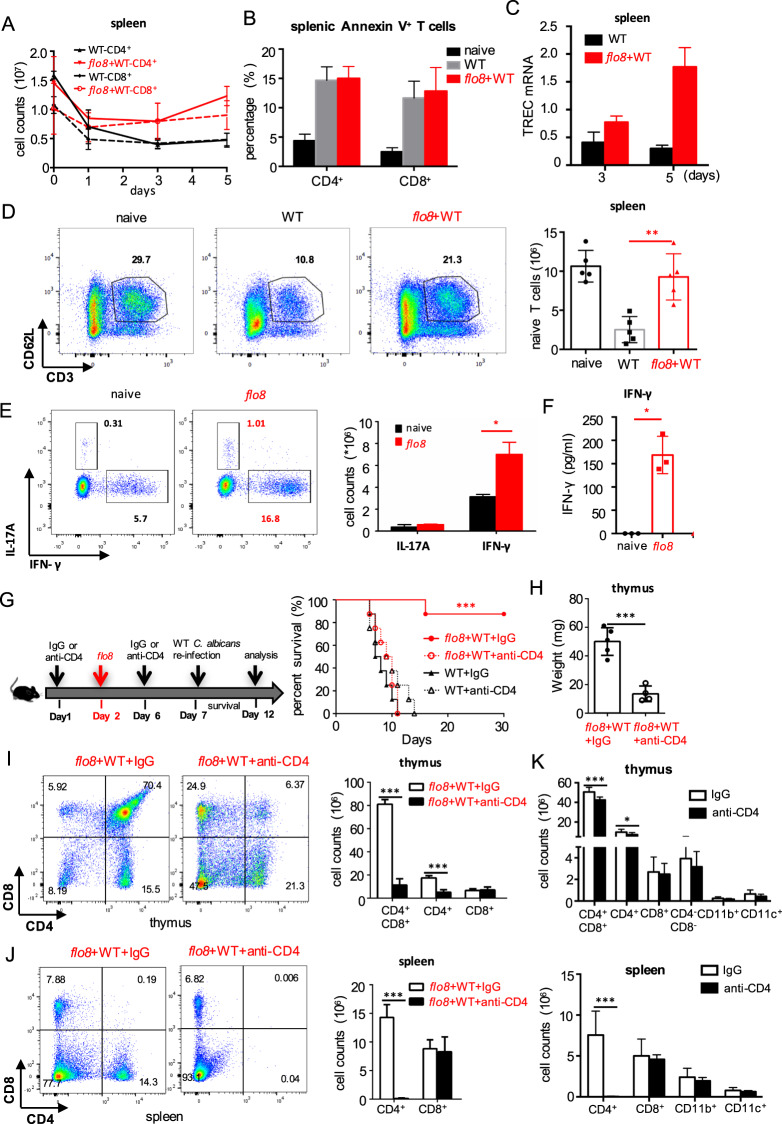
Prior inoculation with the *flo8* mutant promoted naive T-cell supplementation and Th1 differentiation. **A**–**C** Mice were first inoculated with 5 × 10^5^ CFUs of the *flo8* mutant and reinfected with 5 × 10^5^ CFUs of WT *C. albicans* for 3 (**A**, **B**) or 5 (**C**) days (*n* = 5). Flow cytometry was used to analyze the number of CD4^+^ and CD8^+^ T cells (**A**) and the percentages of apoptotic CD4^+^ and CD8^+^ T cells (**B**) in the spleen. RT-PCR was used to determine the expression of the T-cell receptor rearrangement excision circle (TREC) gene in the spleen (**C**). **D** FACS analysis of naive T cells (CD3^+^CD62L^+^ T cells) in the spleens of mice that were previously infected with the *flo8* mutant or not and reinfected with 5 × 10^5^ CFUs of WT *C. albicans* for 5 days. **E** FACS analysis of Th1 and Th17 cells in the spleen. The counts of CD4^+^IL-17A^+^ and CD4^+^IFN-γ^+^ T cells are shown in the right panel. **F** ELISA results for the detection of cytokine IFN-γ secretion from splenic cells of mice that were previously inoculated with *flo8* for 7 days or not. **G**–**J** Strategy (left) for treating mice with anti-mouse CD4 antibodies. The survival curves (right) of mice that were injected *i.p*. with 250 µg of anti-CD4 antibodies prior to infection with 5 × 10^5^ CFUs of the *flo8* mutant and reinfection with 5 × 10^5^ CFUs of WT *C. albicans* (*n* = 8). The thymic weights of the mice were recorded (**H**), and the percentages and numbers of thymic (**I**), and splenic (**J**) T cells were analyzed by flow cytometry. **K** FACS analysis of immune cells in the thymuses and spleens from mice that were treated with 250 µg of anti-mouse CD4 antibodies or with IgG alone for 7 days. Bars, mean ± SEM. *p < 0.05, **p < 0.01, ***p < 0.001, ns not significant, determined by the unpaired *t* test (**B**, **D**, **E**, **F**, **H**, **I**, **J**, **K**). Similar results were obtained in at least two independent experiments

It has been shown that at least half of the peripheral T lymphocytes in the spleen have a 24–48 h life-span, and the renewal of peripheral T cells in the spleen is maintained by the thymic output of mature T cells.^24^ We confirmed that *C. albicans* infection dramatically decreased the CD4^+^ and CD8+ T-cell numbers in the spleen over time (Figs. 3A and S3A). However, priming with the *flo8* mutant successfully maintained the numbers of CD4^+^ and CD8+ T cells in the spleens of infected mice (Figs. 3A and S3A). However, this priming failed to inhibit the apoptosis of splenic CD4^+^ and CD8+ T cells (Figs. 3B and S3B), suggesting that the maintenance of T-cell numbers in the spleen was not attributable to the inhibition of T-cell apoptosis. Since the maintenance of peripheral T cells in the spleen is supported by thymic output,^25^ we assumed that priming with the *flo8* mutant facilitated the output of naive T cells from the thymus to the spleen. We found that priming with the *flo8* mutant increased the expression of splenic T-cell receptor excision circles (TRECs) (Fig. 3C), which are well-established markers for assessing thymic output.^26^ As expected, *C. albicans* infection significantly decreased the number of naive T cells (defined herein as CD3^+^CD62L^+^) in the spleen, while priming with the *flo8* mutant sustained the number of naive T cells in the spleen (Figs. 3D and S3C). Consequently, priming with the *flo8* mutant significantly increased the number of *C. albicans*-induced IFN-γ-producing Th1 cells and the concentrations of IFN-γ in the spleen (Figs. 3E, F and S3D). Together, these data indicated that priming with the *flo8* mutant facilitated the output of naive T cells from the thymus to the spleen, which enhanced *C. albicans*-induced Th1-mediated antifungal immune responses.

We further depleted CD4+ T cells in mice during priming with the *flo8* mutant. We found that intravenous injection of a CD4-specific antibody completely blocked the protective roles of *flo8* mutant priming against *C. albicans* infections (Fig. 3G). Moreover, treatment with anti-CD4 in mice infected with *C. albicans* significantly inhibited thymus weight loss and increased the numbers of CD4^+^CD8^+^ and CD4^+^T cells in the thymus or CD4^+^T cells in the spleen induced by *flo8* mutant priming (Fig. 3H–J). In contrast, this treatment had no influence on the number of CD8+ T cells in the thymus or spleen (Fig. 3H–J). However, treatment with anti-CD4 in uninfected mice decreased the number of CD4+ T cells in only the spleen (Fig. 3K). Thus, these data implied that CD4+ T cells were essential for the protective benefits induced by the *flo8* mutant against *C. albicans* infection.

### Priming with the *flo8* mutant induces IL-10 secretion in the thymus to inhibit thymocyte apoptosis

**Fig. 4:**
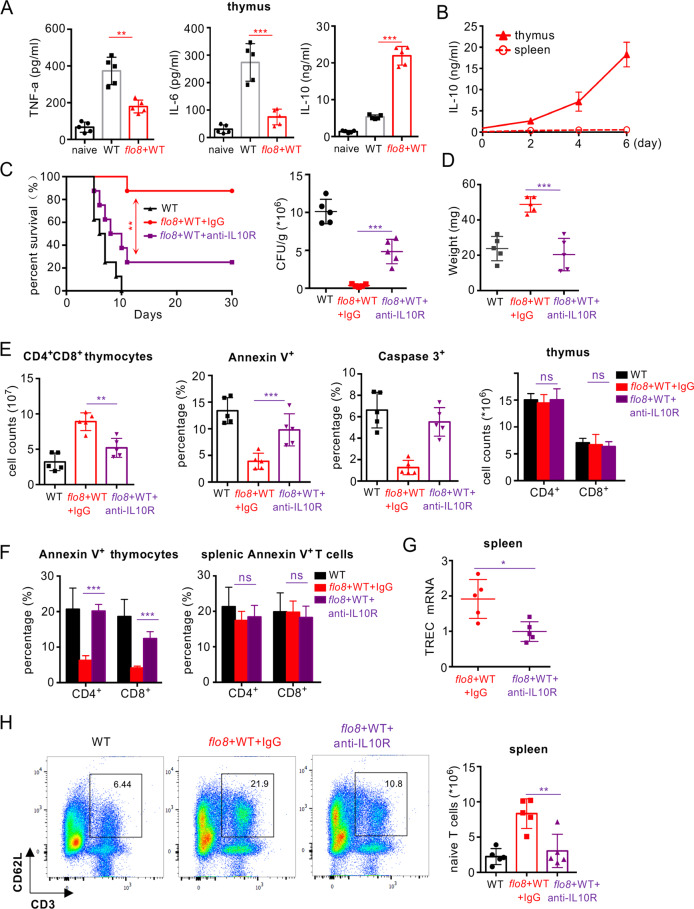
Prevention of thymus atrophy by *flo8* mutant priming was dependent on IL-10 in vivo. **A** ELISA results for the detection of cytokines TNFα, IL-6, and IL-10 in the thymus organs of mice primarily infected with 5 × 10^5^ CFUs of the *flo8* mutant and reinfected with 5 × 10^5^ CFUs of WT *C. albicans* for 24 h. **B** ELISA results of the detection of IL-10 in the thymus and spleen organs of mice infected with 5 × 10^5^ CFUs of the *flo8* mutant for 0–6 days (*n* = 5). **C**–**H** Mice were infected with 5 × 10^5^ CFUs of the *flo8* mutant for 7 days and reinfected with 5 × 10^5^ CFUs of WT *C. albicans*. PBS, rabbit IgG (300 μg/mouse) or an anti-IL10R antibody (300 μg/mouse) was injected intraperitoneally on days 1, 3, 5, 7, and 9. The survival rates and renal fungal burdens of mice were determined (**C**). The weights of the thymus were recorded (**D**). The numbers of thymic T cells and percentages of Annexin V^+^ and caspase3^+^ subpopulations gated in CD4^+^CD8^+^ T cells (**E**) and the percentages of apoptotic CD4^+^ and CD8^+^ T cells in the thymus and spleen (**F**) were analyzed by flow cytometry. The expression of the T-cell receptor rearrangement excision circle (TREC) in the spleen (**G**) was analyzed by RT-PCR. Naive T cells in the spleen were analyzed by flow cytometry (**H**). Bars, mean ± SEM. *p < 0.05, **p < 0.01, ***p < 0.001, ns not significant, determined by the log rank (Mantel–Cox) test (**C**) and the unpaired *t* test (**A**, **C**, **D**, **E**, **G**, **H**, **I**). Similar results were obtained in at least two independent experiments

It has been shown that the anti-inflammatory cytokine IL-10 can suppress thymocyte apoptosis partially by upregulating Bcl-2 expression.^27–29^ Here, we found that *C. albicans* infection significantly increased the concentrations of proinflammatory cytokines, including TNF-α and IL-6, in the thymus (Fig. 4A), whereas priming with the *flo8* mutant significantly decreased the concentrations of these cytokines in the secondary challenge with *C. albicans* (Fig. 4A). However, priming with the *flo8* mutant for 6 days induced IL-10 production at a concentration of ~20 ng/ml in the mouse thymus (Fig. 4B). In contrast, challenge with the *C. albicans* WT strain or *efg1/cph1* mutant for 6 days induced only ~5 ng/ml IL-10 in the mouse thymus (Fig. S4A). These data suggested that the *flo8* mutant was more potent at inducing IL-10 production in the mouse thymus than the WT or *efg1/cph1* mutant. However, no significant changes in IL-10 production were observed in the spleens of the infected mice (Figs. 4B and S4A). To determine whether the thymus protection induced by *flo8* mutant priming was dependent on IL-10, we blocked the function of the IL-10 receptor (IL-10R) using its specific antibody. Blockade of IL-10R significantly impaired the protection induced by *flo8* mutant priming, as determined by a lower survival rate, higher kidney fungal load, and more thymus weight loss during *C. albicans* infection (Fig. 4C, D). Moreover, blocking IL-10R significantly reduced the survival of mice infected with the *C. albicans* WT strain (Fig. S4B), suggesting that the IL-10/IL-10R axis is required for host immune defenses against *C. albicans* infections. Furthermore, this blockade completely inhibited the increases in thymic CD4^+^CD8^+^, CD4^+^, and CD8^+^ T-cell numbers and the frequency of thymic T cells expressing Annexin V induced by *flo8* mutant priming (Figs. 4E, F and S4C, D). However, this blockade had no influence on the frequency of splenic CD4+ T and CD8+ T cells expressing Annexin V (Figs. 4F and S4D). Consistently, blockade of IL-10R completely impaired the *flo8* mutant-induced suppression of the downregulation of the apoptotic genes GILZ and Bim and the upregulation of the antiapoptotic genes Bcl2 and BCL-XL in the thymus (Fig. S4E). Consequently, this blockade also impaired the *flo8* mutant-induced upregulation of TREC expression and the increase in naive T-cell numbers in the spleen during *C. albicans* infection (Fig. 4G, H). Collectively, these data indicated that priming with the *flo8* mutant-induced IL-10 secretion in the thymus to inhibit thymocyte apoptosis and thus confer host protection against *C. albicans* infection.

Furthermore, a low dose of recombinant IL-10 (rIL-10, 1 μg/mouse) significantly decreased the kidney fungal burden in mice infected with WT strains (Fig. S4F). The medium dose of rIL-10 (5 μg/mouse) had no influence on the kidney fungal burden in infected mice (Fig. S4F). However, a high dose of rIL-10 (5 μg/mouse) significantly increased the fungal burden in the kidneys of infected mice (Fig. S4F). Different doses of recombinant IL-10 are known to have differential influences on the survival of mice with sepsis.^30,31^

### Mannans on the surface of the serum-induced *flo8* mutant stimulate macrophages and DCs to produce IL-10

**Fig. 5:**
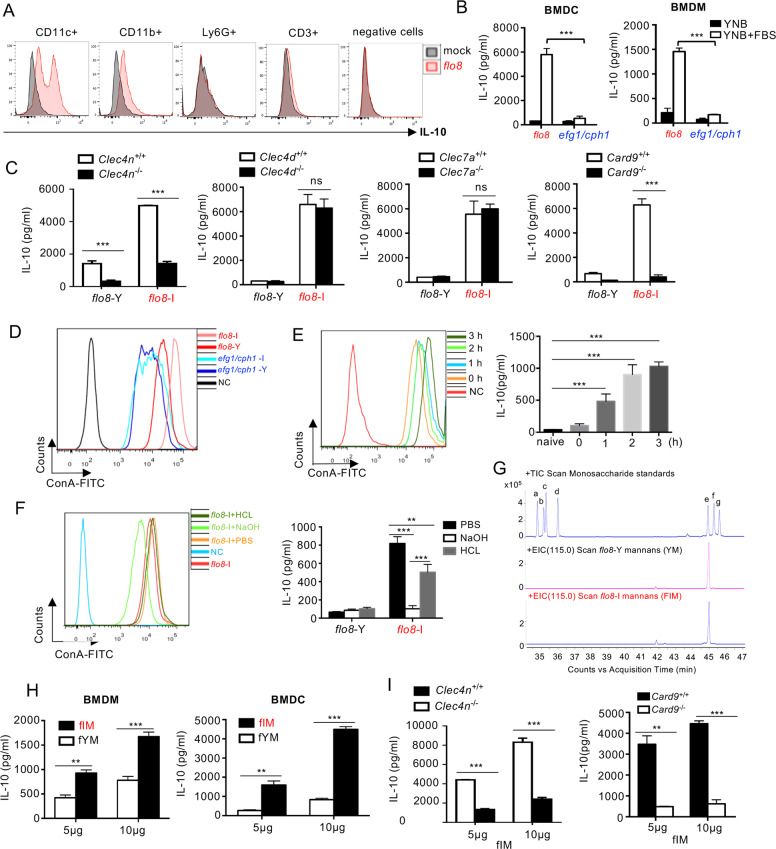
Mannans extracted from the serum-induced *flo8* mutant-induced high levels of IL-10. **A** Intracellular IL-10 staining of cells in thymus organs from mice that were previously inoculated with 5 × 10^5^ CFUs of the *flo8* mutant for 7 days. **B** ELISA results of the detection of IL-10 in BMDMs and BMDCs stimulated with the *C. albicans flo8* or *efg1/cph1* mutant and cultured in YNB at 30 °C or induced with YNB + 10% FBS at 37 °C for 3 h in 5% CO_2_. **C** ELISA results of the detection of IL-10 in wild-type, *Clec4n*^−/−^, *Clec4d*^−/−^, *Clec7a*^−/−^, and *Card9*^−/−^ BMDCs stimulated with the *C. albicans flo8* mutant and cultured in YNB at 30 °C (*flo8*-Y) or induced with YNB + 10% FBS + 5% CO_2_ at 37 °C for 3 h (*flo8*-I). **D** FACS quantification of mannans in *flo8* and *efg1/cph1* mutants cultured in YNB at 30 °C (Y) or induced with YNB + 10% FBS at 37 °C for 3 h in 5% CO_2_ (*flo8*-I). **E** FACS quantification of mannans (left) in the *flo8* mutant induced with YNB + 10% FBS for 0, 1, 2, or 3 h at 37 °C in 5% CO_2._ ELISA results of the detection of IL-10 (right) in BMDMs stimulated with the *flo8* mutant induced for 0, 1, 2, or 3 h (MOI = 5) for 16 h. **F** FACS quantification of mannans in the serum-induced *flo8* mutant treated with acid or alkali (left). The *flo8* mutants were induced with YNB + 10% FBS at 37 °C for 3 h in 5% CO_2_ (*flo8*-I) and heated in PBS, 100 mM HCl or 2% (m/v) NaOH at 95 °C for 15 min. ELISA results of the detection of IL-10 (right) in BMDMs stimulated by the *flo8*-I mutant treated with acid or alkali for 16 h (MOI = 5). **G** Monosaccharide composition analysis of mannans extracted from the *flo8* mutant by GC-MS: monosaccharide standards, a, rhamnose; b, fucose; c, arabinose; d, xylose; e, mannose; f, glucose; g, galactose. **H** ELISA results of the detection of IL-10 in BMDMs and BMDCs stimulated with mannans extracted from the *flo8* yeast mutant (fYM) and serum-induced *flo8* mutant (fIM). **I** ELISA results of the detection of IL-10 in *Clec4n*^−/−^ and *Card9*^−/−^ BMDCs stimulated by fIM. Bars, mean ± SEM. *p < 0.05, **p < 0.01, ***p < 0.001, ns not significant, determined by the unpaired *t* test (**B**, **C**, **E**, **H**, **I**) or one-way ANOVA (**F**). Similar reults were obtained in at least two independent experiments

To explore the source of IL-10 production after priming with the *flo8* mutant, thymocytes from mice primed for 6 days were subjected to immunocytochemical staining to determine the IL-10-producing cells. Thymocytes from mice primed with the *flo8* mutant had a greater frequency of IL-10-producing DCs (defined herein as live CD11c^+^IL-10^+^ cells) and macrophages (defined here as live CD11b^+^IL-10^+^ cells) than unprimed mice (Fig. 5A). However, infections with *flo8*, *efg1/cph1*, and low-dose WT *C. albicans* slightly increased the percentage of Treg (defined herein as live CD4^+^Foxp3^+^) cells in the mouse spleen but had no influence on thymic Treg differentiation (Fig. S5A). Consequently, the frequency of IL-10-producing Treg cells in the thymus organs of mice infected with *flo8*, *efg1/cph1*, or WT *C. albicans* was low (Fig. S5B).

A direct in vitro assay revealed that the serum-induced *flo8* mutant (*flo8*-I) could stimulate higher levels of IL-10 production than the yeast *flo8* mutant (*flo8*-Y) or *efg1/cph1* mutant in BMDCs and BMDMs (Fig. 5B). Furthermore, the deficiency of either Dectin-2 or CARD9, but not Dectin-1 or Dectin-3, completely impaired IL-10 production in BMDCs after stimulation with a serum-induced *flo8* mutant (*flo8*-FI) (Fig. 5C). Thus, these data implied that serum stimulation might increase the exposure of mannans on the surface of the *flo8* mutant to induce Dectin-2/CARD9-mediated IL-10 production in macrophages and DCs.

It has been shown that the structure and content of mannans in *C. albicans* are dynamically altered during dimorphic transition in response to serum induction.^32,33^ We explored the surface quantity of mannans with ConA staining in the *flo8* or *efg1/cph1* mutant with or without serum treatment, and the resulting cells were assayed by flow cytometry as described previously.^34^ As expected, more mannans were exposed on the surface of the serum-induced *flo8* mutant than on those of the *flo8* and serum-induced *efg1/cph1* mutants (Fig. 5D). Furthermore, the quantity of surface mannans in the *flo8* mutant was increased by serum treatment in a time-dependent manner (Fig. 5E). Consistently, the increase in IL-10 production in BMDMs induced by the *flo8* mutant also corresponded to the serum treatment time (Fig. 5E). Moreover, treatment of the serum-induced *flo8* mutant with sodium hydrate successfully removed surface mannans and blocked IL-10 production in BMDMs (Fig. 5F). However, hydrochloric acid treatment had a slight influence on surface mannans in the serum-induced *flo8* mutant and on IL-10 production in BMDCs (Fig. 5F). Thus, these data implied that serum stimulation increased the exposure of mannans on the surface of the *flo8* mutant to induce IL-10 production in macrophages and DCs.

To further confirm our above conclusion, we extracted polysaccharides from the surfaces of WT, *efg1/cph1*, and *flo8* mutants with or without serum induction and then performed hydrolysis reactions to convert the polysaccharides to monosaccharides for GC-MS analysis. Monosaccharide composition analysis showed that the polysaccharides from the *flo8* mutant (fYM) and serum-induced *flo8* mutant (fIM) contained mainly mannose (Fig. 5G), indicating that the extracted polysaccharides primarily comprised mannans. As expected, the mannans extracted from the serum-induced *flo8* mutant (fIM) were more potent than those extracted from the WT (WYM and WIM), *efg1/cph1* (eYM and eIM), and *flo8* mutant (fYM) to induce IL-10 production by BMDMs or BMDCs in a dose-dependent manner (Figs. 5H and S5C). Deficiency of either Dectin-2 or CARD9 completely impaired IL-10 production in BMDCs after stimulation with fIM (Fig. 5I). Collectively, these data confirmed that serum stimulation increased the exposure of mannans on the surface of the *flo8* mutant to induce Dectin-2/CARD9-mediated IL-10 production in macrophages and DCs.

### Priming with mannans extracted from the *flo8* mutant elicits protective immunity against *C. albicans* infection

**Fig. 6:**
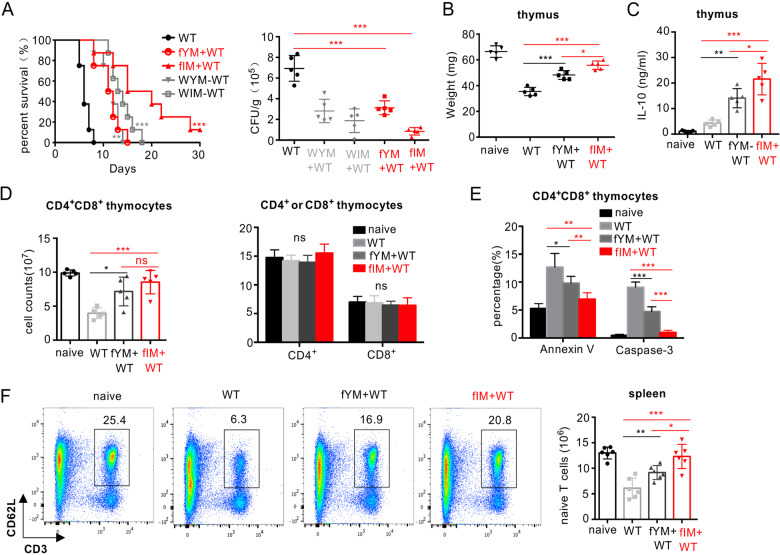
Mannans extracted from the serum-induced *flo8* mutant exert protective effects. **A** Survival curves and renal fungal burdens of mice injected with different mannans extracted from yeast *flo8* mutant (fYM), yeast WT strains (WYM), serum-induced *flo8* mutant (fIM), and serum-induced WT (WIM) strains. **B**–**F** C57BL/6 mice were injected *i.p*. with PBS or fYM, fIM WYM, WIM (1 mg/mouse) at days 1, 3, 5, and 7 and infected with 3 × 10^5^ CFUs of WT *C. albicans* at day 8. The weights (**B**) and IL-10 protein levels (**C**) of the thymus were determined. The numbers of T-cell subpopulations (**D**), the percentages of Annexin V^+^ and caspase 3^+^ CD4^+^CD8^+^ cells (**E**) in the thymus at day 10 and the number of naive T cells (**F**) in the spleen at day 14 were analyzed by flow cytometry. Bars, mean ± SEM. *p < 0.05, **p < 0.01, ***p < 0.001, ns not significant, determined by the log rank (Mantel–Cox) test (**A**) or one-way ANOVA (**B**, **C**, **D**, **E**, **F**). Similar results were obtained in at least two independent experiments

We further explored whether mannans extracted from WT strains (WYM vs. WIM) and *flo8* mutants with or without serum induction (fIM vs. fYM) elicited immunoprotection against *C. albicans* infection. We observed that priming with fIM potently elicited immunoprotection against *C. albicans* infection compared with that achieved by priming with fYM, WYM, or WIM, as determined by a higher survival rate, lower kidney fungal load, and less thymus weight loss (Fig. 6A, B). Furthermore, priming with fIM or fYM significantly increased the concentrations of IL-10 in the thymus organs of mice infected with *C. albicans* (Fig. 6C). Priming with fIM or fYM successfully maintained the number of CD4+ CD8+ T cells and significantly decreased the frequency of Annexin V and caspase-3 expression in CD4+ CD8+ T cells in the thymuses of mice infected with *C. albicans* (Figs. 6D, E and S6A). Consequently, priming with fIM or fYM sustained the number of naive T cells (defined herein as CD3^+^CD62L^+^) in the spleens of mice infected with *C. albicans* (Fig. 6F). Collectively, these data suggest that mannans extracted from the *flo8* mutant could induce IL-10 production to inhibit *C. albicans*-induced thymus atrophy by blocking the apoptosis of immature CD4+ CD8+ T cells.

### Priming with the *flo8* mutant induces protective immunity against polymicrobial sepsis

**Fig. 7:**
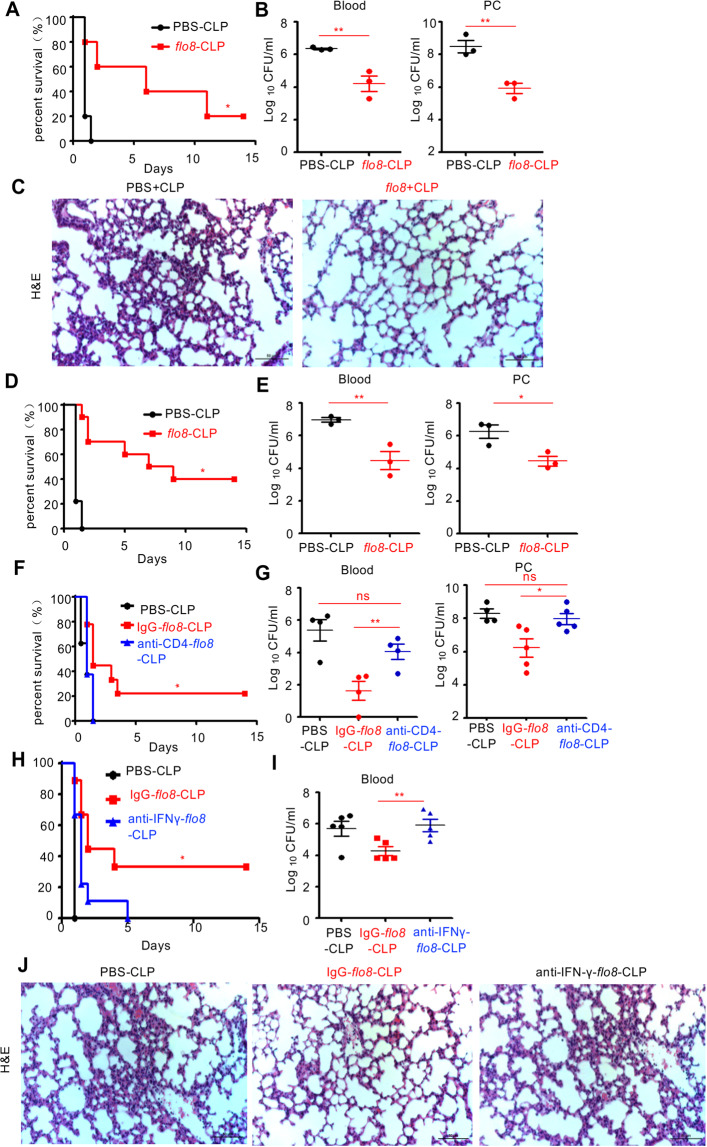
Pre-exposure to the *flo8* mutant protects mice from subsequent lethal polymicrobial sepsis caused by CLP. **A**–**C** Mice were preinfected with 5 × 10^5^ CFUs of the *flo8* mutant or with PBS for 28 days and then subjected to CLP. The survival curves (**A**) and microbial burdens in the blood or peritoneal cavity (PC) were determined (**B**), and H&E-stained sections of lungs from septic mice induced by CLP are shown. **D**, **E** Mice in the *flo8* pre-exposure group were preinfected with 5 × 10^5^ CFUs of the *flo8* mutant or with PBS for 7 days and then subjected to CLP. The survival curves (**D**) and microbial burdens in the blood or peritoneal cavities (PCs) (**E**) of septic mice induced by CLP were determined. **F**, **G** Mice preinfected with the *flo8* mutant were injected *i.p*. with 200 µg of IgG or anti-mouse CD4 antibodies 1 day before *C. albicans* infection. The survival curves (**F**) and microbial burdens in the blood or peritoneal cavities (PCs) (**G**) of mice were determined. **H**–**J** Mice preinfected with the *flo8* mutant were injected *i.p*. with 200 µg of IgG or anti-mouse IFN-γ antibodies on the day of *C. albicans* infection and at 3-day intervals thereafter until the completion of the experiment. Survival curves (**H**), microbial burdens in the blood (**I**), and H&E-stained sections of lungs (**J**) from septic mice induced by CLP are shown. *n* = 8–10 for the survival curves, and *n* = 3–5 for the microbial burden. Bars, mean ± SEM. *p < 0.05, **p < 0.01, ***p < 0.001, PC peritoneal cavity

To explore whether priming with the *flo8* mutant had protective effects against lethal polymicrobial infections, we performed cecal ligation and perforation (CLP) to construct a mouse model of sepsis as described previously.^35^ Interestingly, priming with the *flo8* mutant for a 28-day interval resulted in increased survival and a decreased bacterial burden in the blood and peritoneal cavities of CLP-induced septic mice (Fig. 7A, B). Consistently, this priming significantly reduced CLP-induced tissue lesions, with thinner alveolar septa and less inflammatory cell infiltration than those in control mice (Fig. 7C). In addition, priming with the *flo8* mutant for a 7-day interval also significantly protected mice from CLP-induced sepsis, as shown by increased survival and a decreased bacterial burden in the blood and peritoneal cavity (Fig. 7D, E). However, this priming provided no protection for LPS-induced septic mice, which may have been due to the higher levels of proinflammatory cytokines induced by both the *flo8* mutant and LPS in mice (Fig. S7A–C). We further primed athymic nude mice and *RAG1*-deficient mice with CLP-induced sepsis. Priming with the *flo8* mutant provided no protection against lethal CLP-induced sepsis in *RAG1*-deficient or nude mice (Fig. S7D–G). These data indicate that the thymus is essential for protective responses induced by the *flo8* mutant against lethal polymicrobial infections.

We further explored whether Th1-mediated responses were essential for *flo8* mutant-induced protection against polymicrobial infections. We found that intravenous injection of a CD4- or IFN-γ-specific antibody completely blocked the protective effects of *flo8* mutant priming against CLP-induced sepsis, as shown by lower survival rates and increased bacterial burdens in the blood and peritoneal cavity compared with those of primed mice receiving control IgG (Fig. 7F–I). Moreover, neutralization of IFN-γ in primed mice with CLP-induced sepsis significantly aggravated lung tissue damage (Fig. 7J). Thus, these data indicated that the Th1-mediated responses induced by *flo8* mutant priming were critical for immunoprotection against lethal polymicrobial infections.

## Discussion

The relationship between virulence and morphological transformation is the key issue in studying the pathogenicity of *C. albicans*.^10,36^ In this study, we identified *FLO8* as a potential immunotherapeutic target against systemic *C. albicans* infection. The disruption of *FLO8* in *C. albicans* could inhibit the yeast-to-hyphae transition and elicit protective immune responses against systemic candidiasis in a murine model. Furthermore, the immune protection induced by the *flo8* null mutant is not the commonality of all hyphae-deficient strains since the *efg1/cph1* null mutant kept in yeast form provided no immunoprotection against subsequent lethal *C. albicans* infection. However, a genetically engineered *C. albicans tet-NRG1* strain, which is conditionally kept in yeast form in vivo, can evoke a protective immune response against subsequent lethal *C. albicans* infection.^37^ The *C. albicans* strain PCA-2, which is incapable of yeast-to-hyphae conversion, confers protection against subsequent challenge with a highly pathogenic *C. albicans* strain.^38,39^ However, the yeast cell-associated molecules that trigger protective immune responses remain unclear. During the course of *C. albicans* infection, the initial response of the innate immune system is determined by the recognition of fungal cell wall components, including glucans and mannans.^40,41^ A recent study showed that fungus-derived mannans, but not glucan-containing curdlan and zymosan, mediate the protective benefits of commensal fungi.^6^ In our study, we showed that mannans extracted from serum-induced *flo8* mutants could elicit immunoprotection against subsequent lethal *C. albicans* infection. In contrast, mannans extracted from the *flo8* mutant had a slight immunoprotective effect. Therefore, mannans exposed on serum-induced *flo8* mutant cells may be critical for evoking protective immune responses against invasive candidiasis or candidemia in a murine model, and serum induction may facilitate mannan modification, which is negatively regulated by *FLO8* in *C. albicans*.^33^ Further studies are needed to clarify the structural alterations of mannans induced by serum in *flo8* mutants.

Immunological studies on infectious diseases have focused mainly on the effector immune response, changes in the blood and peripheral lymphoid organs of infected individuals, and vaccine development.^42,43^ Studies on the thymus in infected individuals have been neglected despite that the thymus is a primary lymphoid organ in which bone marrow-derived T-cell precursors undergo differentiation, ultimately leading to the migration of positively selected thymocytes to the T-cell-dependent areas of peripheral lymphoid organs.^44^ Recent studies show that normal thymocyte development and export can be altered as a result of different infectious diseases caused by viruses, protozoa, and fungi.^20^ One common feature is the severe atrophy of the infected thymus, mainly due to the apoptosis-related depletion of immature CD4+ CD8+ T cells.^21^ As expected, our study showed that systemic infection with *C. albicans* caused severe thymus atrophy by inducing the apoptosis of immature CD4^+^CD8^+^ and mature CD4^+^ or CD8^+^T cells. During normal thymocyte development, 80% of the total thymocyte population is CD4+ CD8+ T cells, whereas *C. albicans* infection reduces the percentage of CD4+ CD8+ T cells to ~60% of the whole population. Furthermore, we found that *C. albicans* infection significantly increased the percentage of thymic T cells expressing apoptosis-related markers, including Annexin V and caspase-3, by increasing the expression levels of proapoptosis-related genes (GILZ and Bim) and decreasing the expression levels of antiapoptosis-related genes (Bcl2 and BCL-XL).^27,45^ However, we surprisingly found that priming with the *flo8* null mutant or mannans extracted from serum-induced *flo8* mutant cells could completely block *C. albicans*-induced thymus atrophy by inhibiting the apoptosis of thymic T cells. More importantly, our study showed that the *flo8* mutant or mannans extracted from serum-induced *flo8* mutant cells could induce high levels of the anti-inflammatory cytokine IL-10 to increase the expression of Bcl2 and BCL-XL and decrease the expression of GILZ and Bim, ultimately resulting in decreased percentages and numbers of Annexin V- and caspase-3-expressing T cells. Conversely, blockade of IL-10R using its specific antibody or deficiency in the mouse thymus could completely inhibit the protective immune responses induced by the *flo8* mutant or mannans extracted from serum-induced *flo8* mutant cells. Recent studies have demonstrated that IL-10 can suppress lymphocyte apoptosis partially by upregulating Bcl-2 expression, which is associated with the improved survival of septic mice and indicates that prevention of thymocyte apoptosis may contribute to the advantageous outcome and be a potential therapeutic target.^27,28^ It is difficult to evaluate the direct effect of thymus atrophy on the immune system, but a previous study reported that newly activated cells migrating from the thymus are critical for the sustainment of antiviral immunity.^46^ In addition, thymic atrophy caused by chronic virus infection impairs negative selection to facilitate the escape of self-reactive T cells.^47^

Sepsis is the systemic inflammatory response syndrome caused by bacterial, viral, or fungal infections.^1^ Although hundreds of clinical trials have been conducted, no effective new therapies against sepsis have been approved. Priming with live attenuated vaccines or commensal microorganisms is increasingly being recognized to improve many aspects of the host immune system.^4–6,48–50^ Here, we show that prior inocuation of *FLO8*-deficient *C. albicans* could effectively protect mice from polymicrobial sepsis induced by CLP. The protection depends on Th1-mediated adaptive immune responses. The immunopotentiating effect of the *flo8* mutant makes it a live attenuated vaccine capable of preventing polymicrobial infections.

Collectively, our data imply that Flo8p, a key transcription factor for regulating the yeast-to-hyphae transition of *C. albicans*, has potential to be both an antifungal and immunotherapeutic target, and mannans extracted from the cell wall of the *flo8* null mutant strain may provide potential immunotherapeutic candidate(s) for controlling fatal infectious diseases.

## Materials and methods

### Fungi, media and growth conditions

*C. albicans* strains SC5314 (WT), UCA3, UCA21, *flo8* (*FLO8Δ/Δ*), *flo8* + *FLO8* (*FLO8Δ/FLO8*), and *efg1/cph1* (*EFG1Δ/ΔCPH1Δ/Δ*) were grown overnight in YPD-rich medium at 30 °C. UV- and heat-killed *C. albicans* strains were exposed to 1000 J/cm^2^ of UV 5 times in a CL-1000 cross-linker and to a 95 °C water bath for 10 min, respectively. *C. albicans* yeast cells were incubated with YNB plus 10% FBS at 37 °C for 3 h. The *C. albicans* strains used in this study are listed in Table S1.

### Preparation of BMDMs and BMDCs

For BMDM preparation, bone marrow cells isolated from 6- to 8-week-old female mice were cultured in medium containing M-CSF medium, 10% FBS and 30% of the culture supernatant from L929 cells. Fresh M-CSF-containing medium was added on day 3, and the fully differentiated BMDMs were harvested on day 6 for the functional assay.

BMDCs were generated by cultivating murine bone marrow cells (1 × 10^6^ cells/ml) from 6- to 8-week-old female mice in growth medium supplemented with recombinant M-CSF (40 ng/ml). The culture medium was replaced every 2 days, and the fully differentiated DCs were harvested on day 8 for the functional assay.

### Mice

C57BL/6, Balb/c, and nude mice were purchased from Shanghai SLAC Laboratory Animal Co, Ltd. *Clec7a*^−/−^, *Clec4n*^−/−^, *Clec4e*^−/−^, *Card9*^−/−^, and *Rag1*^−/−^ mice were maintained at Tongji University Animal Center. All mice were genotyped by PCR using genomic tail DNA. All mice were housed under specific pathogen-free conditions at Tongji University. All animal experiments were performed in compliance with institutional guidelines and according to the protocol approved by the Institutional Animal Care and Use Committee of Tongji University.

### Primary infection and reinfection models

Six- to eight-week-old female mice were challenged *i.v.* with live or dead *flo8* mutant strains at 2.5 × 10^6^ CFUs/ml. After 7 or 14 days of inoculation, the mice were reinfected *i.v*. with the same dose of the *flo8* mutant, WT *C. albicans* SC5314, or clinically isolated UCA3 or UCA21 strain. Survival of the reinfected mice was observed for 30 days. For the immunoprotection of mannans, mice infected with WT *C. albicans* SC5314 strains were injected intraperitoneally with 1 mg of mannans extracted from *flo8* mutant strains at days 1, 3, 5, and 7. For IL-10R blocking, mice inoculated with 5 × 10^5^ CFUs of *flo8* mutant cells were injected intraperitoneally with 300 μg of anti-CD210 antibodies or IgG at days 1, 3, 5, and 7. Then, the mice were reinfected *i.v*. with 5 × 10^5^ CFUs of WT *C. albicans* SC5314 strains and received 300 μg of anti-CD210 antibodies at day 8. On day 9, the mice were sacrificed, and thymocytes were collected for flow cytometry and RT-PCR analysis. For CD4^+^ T-cell depletion, mice were injected *i.p*. with 200 µg of an anti-CD4 antibody (clone GK1.5) one day before *C. albicans* infection. For IFN-γ neutralization, mice were injected *i.p*. with 200 µg of an anti-IFN-γ antibody (clone XMG1.2) on the day of *C. albicans* infection and reinjected at 3-day intervals until the completion of the experiment.

### Sepsis model

The sepsis model was generated by injection of 10 mg/kg LPS (*i.p*.) or cecum ligation and puncture (CLP) as described previously.^35^ Briefly, mice were anesthetized with 1% pentobarbital solution, and a midline abdominal incision was performed. The ileocecal valve was ligated on the distal 3/4 end of the cecum. The cecum was perforated by a through and through puncture with an 18 G needle. A feces droplet was extruded from the hole to induce polymicrobial sepsis. The abdominal wall was sutured, and the mice were injected *s.c*. with 1 ml of 0.9% NaCl for fluid resuscitation.

### Recoverable microbial burden

The viable microorganisms in infected organs were determined by counting the number of colony-forming units (CFUs). The spleen, liver, or kidneys were harvested from mice infected with *C. albicans*. The homogenized organs were diluted with PBS and plated on SDA agar. CFUs were counted after incubation at 30 °C for 48 h.

Blood and peritoneal fluid from septic mice were collected, diluted, and plated on MHI agar. Microbial burdens were counted after incubation at 37 °C for 24 h.

### Cytokine measurement

The serum and supernatants of homogenized thymus, spleen, and kidney organs from mice infected with *C. albicans* were collected. The supernatants were collected from splenic cells, which were seeded in 96-well U-shaped plates at a density of 1 × 10^6^ cells/well and cultured at 37 °C for 48 h. The protein levels of IL-10, IFN-γ, IL-17A, IL-6, and TNF-α were measured using mouse ELISA kits (eBioscience) according to the manufacturer’s instructions.

BMDMs and BMDCs were stimulated with UV-killed *C. albicans* for 16 h. Then, the supernatants were collected for enzyme-linked immunosorbent assays (ELISAs), which were performed to assess the indicated cytokines according to the manufacturer’s instructions.

### RNA isolation and quantitative reverse transcription PCR

Mice infected with *C. albicans* strains for 24 h were sacrificed. The cells from the thymus were harvested and counted. Total RNA was isolated using 1 ml of TRIzol (Takara) according to the manufacturer’s protocol. RNA (500 ng) was reverse transcribed with PrimeScript™ RT Master Mix. RT-PCR was performed using SYBR^®^ Premix Ex Taq™ II. GAPDH served as the housekeeping gene, and the expression of genes was calculated by ΔΔCt.

### Cell staining and flow cytometry

Single-cell suspensions of thymus or spleen organs harvested from uninfected or infected mice were prepared. For detecting apoptosis, cells were incubated with FITC-CD45, V450-CD4, APC-CD8a, and Annexin V antibodies in the binding buffer at room temperature for 20 min. Then, the cells were washed with PBS + 2% FBS twice and treated with 5 μl of 7-AAD in 100 μl of binding buffer for 15 min. After washing with 500 μl of 1× binding buffer, the cells were filtered into flow tubes and analyzed by BD FACSverse. To detect caspase-3 expression, cells were stained with FITC-CD45, APC-CD8a, and V450-CD4 antibodies. Then, the cells were washed with PBS and incubated with 1 ml of BD Cytofix/Cytoperm solution for 20 min on ice. Subsequently, the cells were stained with PE-caspase3 antibodies at room temperature for 30 min. After washing with 1× Perm/wash buffer, the cells were filtered into flow tubes and analyzed by BD FACSreverse. To detect naive T cells, splenic cells were stained with living dye as well as FITC-CD3, V450-CD4, APC-CD8, and PerCP 5.5-CD62L antibodies on ice for 20 min and washed with PBS + 2% FBS. For detecting Th1 cells, splenic cells were obtained as described above and stimulated with phorbol 12-myristate 13-acetate (PMA, 50 μg/ml) and ionomycin (1 μM) for 5 h in the presence of brefeldin A (5 mg/ml). Surface staining was performed, followed by intracellular staining for IFN-γ or IL-17A using the BD Cytofix/Cytoperm Kit (BD Biosciences). After washing with PBS, cells were filtered into flow tubes and analyzed by BD FACSreverse, and data were analyzed with FlowJo (Tree Star).

### Mannans staining by ConA-Alexa Fluor 48 Conjugate

Yeast *C. albicans* was cultured in YNB medium at 30 °C overnight. Serum-induced *C. albicans* were cultured in YNB + 10% FBS medium at 37 °C for 3 h. After washing three times with PBS, 1 × 10^7^ cells were fixed with 4% paraformaldehyde for 1 h. The cells were washed three times with PBS and stained with 500 μl of 50 μg/ml ConA-Alexa Fluor 48 Conjugate at 30 °C for 1 h. After incubation, the cells were washed five times with PBS and analyzed by BD FACSverse.

### Mannan disruption by acid or alkali

*C. albicans flo8* mutants were cultured in YNB + 10% FBS medium at 37 °C for 3 h in 5% CO_2_. The cells were collected, washed, and counted. A total of 1 × 10^8^ cells were resuspended in PBS, 2% (w/v) NaOH or 10 mM HCl and then heated in a 95 °C water bath for 15 min. Then, the cells were collected for the following experiments.

### Extraction of *C. albicans* mannans

Mannans from *C. albicans* were extracted as previously described.^51^ Briefly, yeast mannans (YM) were extracted from *C. albicans* cultured in YNB medium for 24 h at 30 °C. Induced mannans (IM) were extracted from *C. albicans* grown in YNB + 10% FBS medium for 3 h at 37 °C in 5% CO_2_. *C. albicans* cells (1 g wet weight) were heated in 4 ml of 2% (wt/vol) NaOH solution for 1 h at 100 °C. After centrifugation, the supernatant was collected. Mannans were precipitated from the cold supernatant with fresh Fehling’s reagent. The precipitate was dissolved in a 3 M HCl solution. Subsequently, a solution of methanol and acetic acid mixed at a ratio of 8:1 (vol/vol) was added to the mannnans in the solution. The processes of dissolution and precipitation were performed twice. After that, the precipitate was centrifuged and dissolved in distilled water. Finally, mannans were dialyzed for 24 h in ddH_2_O. The protein levels of purified mannans were less than 0.5%, as determined by the Micro BCA Protein Assay Kit. The endotoxin concentration of extracted mannans was ~0.35 EU/mg, as determined by the ToxinSensor Chromogenic LAL Endotoxin Assay Kit.

### Monosaccharide composition analysis by GC-MS

Monosaccharide standards (d-galactose, d-arabinose, l-rhamnose, l-fucose, d-mannose, d-xylose, and d-glucose) were treated as described previously.^52,53^ Two milligrams of extracted mannans or monosaccharide standards were hydrolyzed with 2 M trifluoroacetic acid at 121 °C for 2 h. After evaporation, the residue was dissolved in 200 μl of H_2_O and reduced with 1 ml of 0.5 mol/l NaBH_4_ DMSO solution at 40 °C for 90 min. Then, glacial acetic acid was added dropwise to the reaction tubes. The residue was acetylated with 200 μl of 1-methylimidazole and 1 ml of acetic anhydride at 40 °C for 30 min to obtain the acetylated derivatives, which were then extracted with 2 ml of chloroform. The chloroform layer was washed three times with ddH_2_O. Subsequently, the chloroform layer was dried by anhydrous sodium sulfate and analyzed by an Agilent Technologies 7890B-5977A GC/MSD system. The temperature profile started at 120 °C and increased to 190 °C at 3 °C/min, followed by a 2 °C/min gradient up to 250 °C, where it was held for 5 min.

## Supplementary information

Supplementary Figures and Tables
