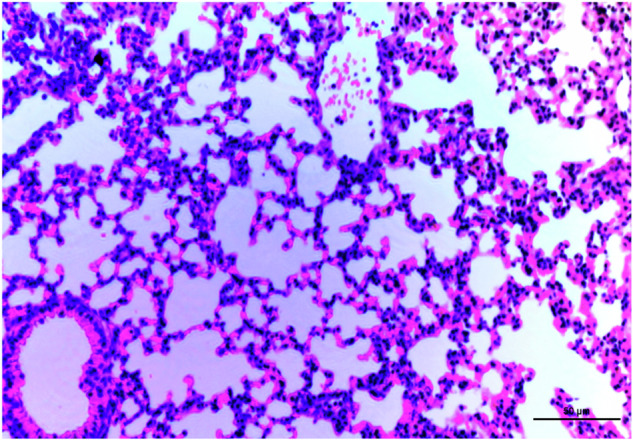# Author Correction: Priming with *FLO8*-deficient *Candida albicans* induces Th1-biased protective immunity against lethal polymicrobial sepsis

**DOI:** 10.1038/s41423-024-01214-1

**Published:** 2024-09-10

**Authors:** Quan-Zhen Lv, De-Dong Li, Hua Han, Yi-Heng Yang, Jie-Lin Duan, Hui-Hui Ma, Yao Yu, Jiang-Ye Chen, Yuan-Ying Jiang, Xin-Ming Jia

**Affiliations:** 1 Clinical Medicine Scientific and Technical Innovation Center, Shanghai Tenth People’s Hospital, Tongji University School of Medicine, Shanghai, 200092 China; 2 School of Pharmacy, Second Military Medical University, Shanghai, 200433 China; 3 State Key Laboratory of Molecular Biology, Shanghai Institutes for Biological Sciences, Chinese Academy of Sciences, Shanghai, 200031 China

Correction to: *Cellular & Molecular Immunology* 10.1038/s41423-020-00576-6, published online 5 November 2020

In this article, which was published online on 5 Nov 2020, an unintended error occurred during the image processing of Fig. 7J. In detail, the image for the control group in Figure 7J (left panel) was inadvertently captured from the same field in the IFN-γ neutralizing group (Figure 7J, right panel). We have re-selected the pathological images of the control group in Figure 7J (left panel) from the raw data and corrected Figure 7J accordingly. The corrected Figure 7 is presented below. The error and correction did not impact the conclusion of the paper. The authors sincerely regret this error. The original article has been corrected.

Figure 7J in the original article:
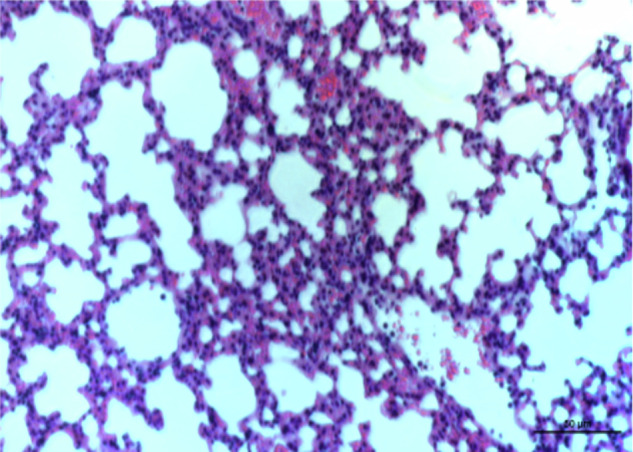


Corrected figure 7J: