# Supplementary material for: Priming with FLO8-deficient Candida albicans induces Th1-biased protective immunity against lethal polymicrobial sepsis
Source: Cell Mol Immunol. 2020 Nov 5;18(8):2010–23. doi: 10.1038/s41423-020-00576-6 (PMC7642578; doi:10.1038/s41423-020-00576-6)
Supplement: Supplementary file 1 — Supplementary Figures and Tables [file 41423_2020_576_MOESM1_ESM.docx]

Lv *et al*. Priming with *FLO8-*deficient *Candida albicans* induces Th1-biased protective immunity against lethal polymicrobial sepsis.

**﻿**

**
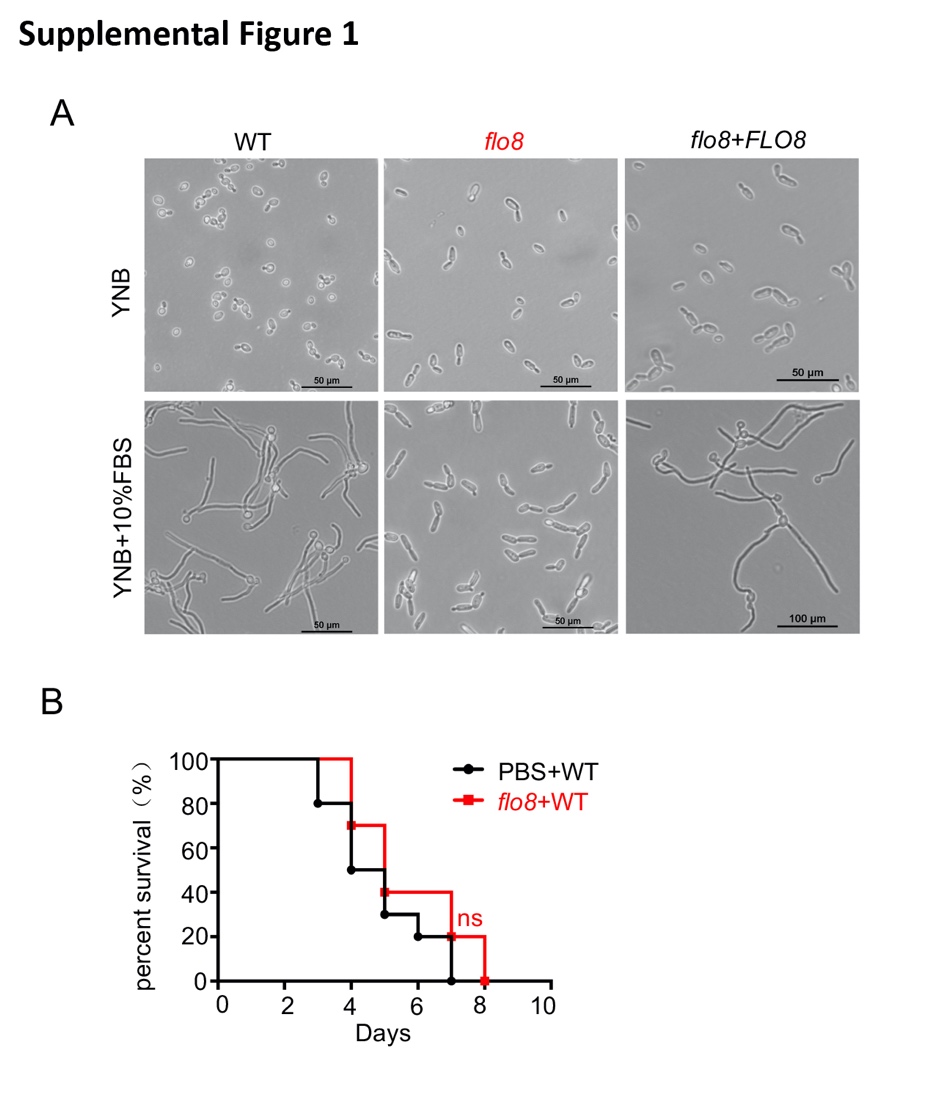
**

**Fig. S1 (A)** Deficient hyphae formation of *flo8* mutant. Representative images of WT *C. albicans*, *flo8* *null* mutant and *FLO8* revertant strains (*flo8*/*FLO8*) cultured in medium YNB+10% FBS+5% CO_2_ at 37℃ for 3 h. **(B)** Survival analysis of mice which were prior inoculated with 5×10^5^ *flo8* mutant for 3 days and reinfected with 5×10^5^ WT *C. albicans*.


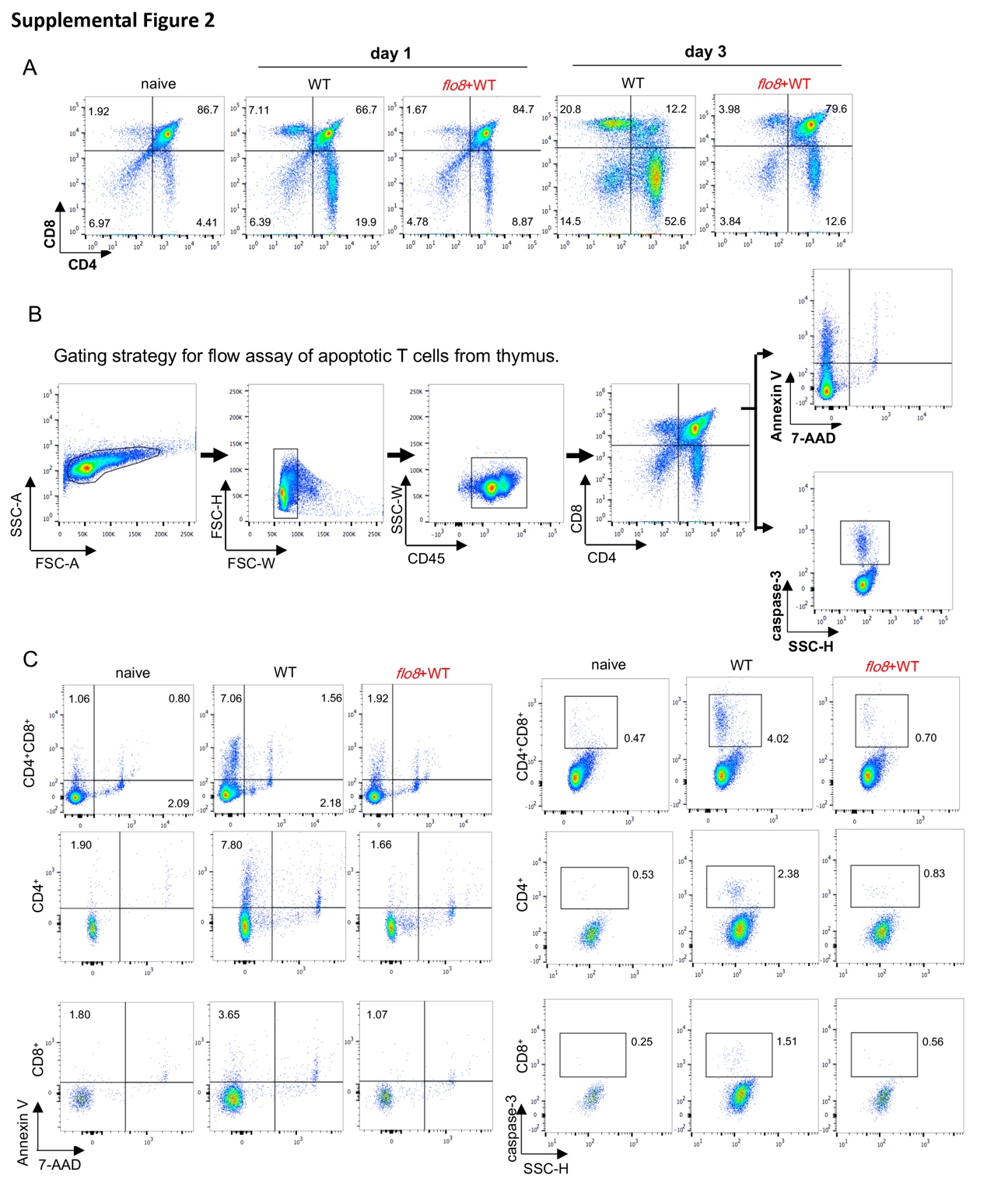


**Fig. S2 (A)** Flow plots represents in Fig. 2E. **(B)** Cell gating strategy for apoptotic T cells in thymus. **(C)** Flow plots represents in Fig. 2F.

**
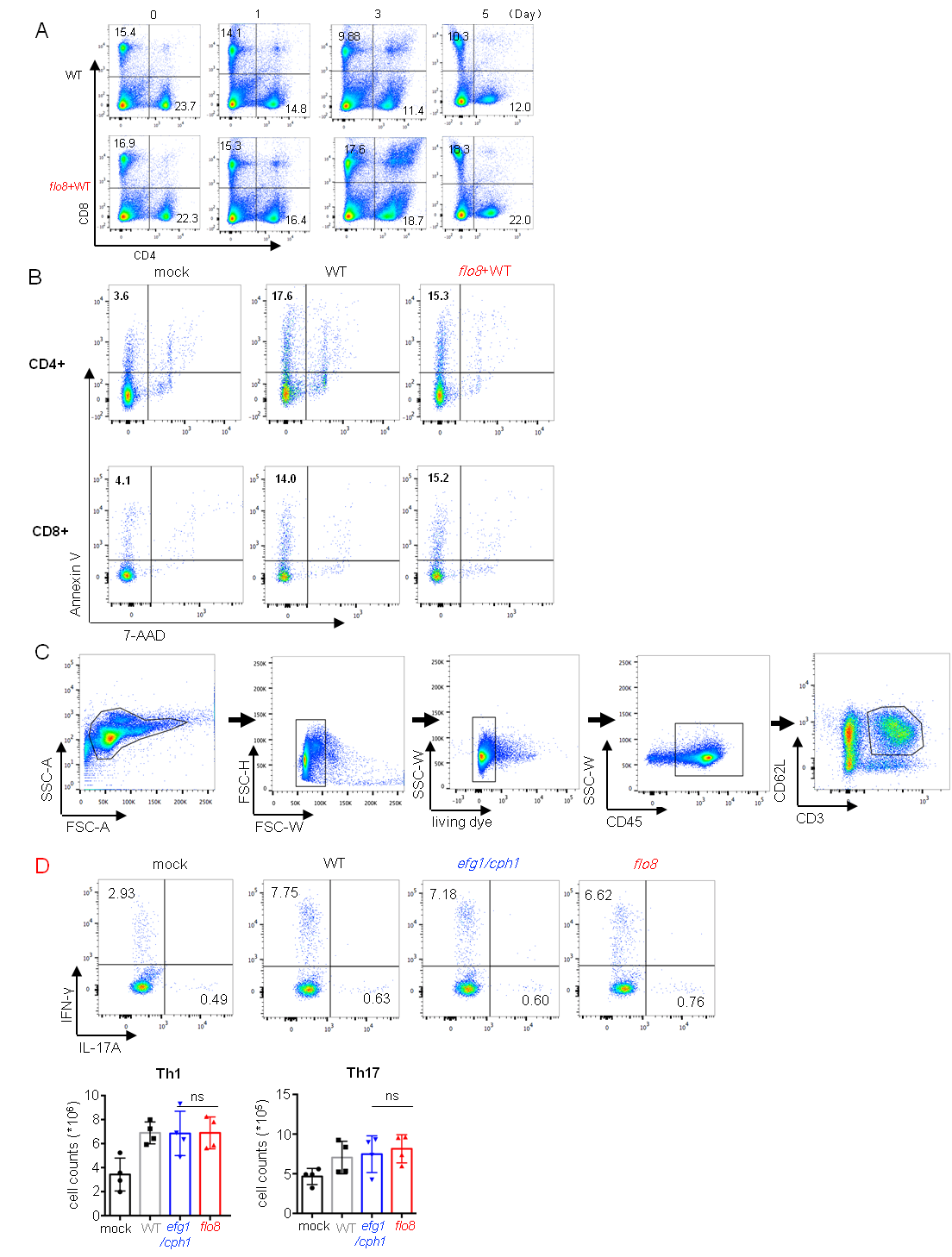
**

**Fig. S3 (A)** Flow plots represents in Fig. 3A. **(B)** Flow plots represents in Fig. 3B. **(C)** Cell gating strategy for naive T cells in spleen. **(D)** FACS analysis of Th1 and Th17 cells in spleen from mice, which were infected with 1×10^5^ WT *C. albicans*, 5×10^5^ *efg1/cph1* or 5×10^5^ *flo8* mutant for 7 days (n=4). The cell counts of CD4^+^IL-17A^+^ and CD4^+^IFN-γ^+^ T cells were showed in the below panel. ns, not significant, by unpaired t test.

**Fig. S4 (A)** ELISA results of IL-10 in thymus from mice which were infected with 1×10^5^ WT *C. albicans*, 5×10^5^ *efg1/cph1* or 5×10^5^ *flo8* mutant for 2, 4 and 6 days (n=3). **(B)** Survival analysis of mice which were infected with 5×10^5^ WT *C. albicans*. Rabbit IgG (300 μg/mice) or anti-IL10R antibodies (300 μg/mice) were injected intraperitoneally at day 1, 3, 5 (n=8). *p < 0.05, by log rank (Mantel-Cox) test**. (C)** Flow plots represents in Fig. 4E**. (D)** Flow plots repressents in Fig. 4F. **(E)** Expression of pro-apoptotic and anti-apoptotic genes in mice treated with IL-10R antibodies, mentioned in Fig 4D. **(F)** Fungal burden in the kidney from mice which were infected with 5×10^5^ WT *C. albicans* (n=3)*.* After 6 hours infection, mice were injected with PBS, 1 μg，2 μg or 5 μg rIL-10 intraperitoneally. **p < 0.01, ***p < 0.001, by unpaired t test.

**Fig. S5 (A)** FACS analysis of Treg cells in thymus and spleen from mice infected with 1×10^5^ WT *C. albicans*, 5×10^5^ *efg1/cph1* or 5×10^5^ *flo8* mutant for 7 days (n=4). The percentage of CD4^+^Foxp3^+^ T cells were showed in the right panel. ns, not significant, by unpaired t test. **(B)** Intracellular IL-10 staining of Treg cells in spleen and thymus. Mice were treated as Fig. S5A. **(C)** ELISA results of IL-10 in BMDMs and BMDCs, which were stimulated with 10 μg mannans extracted from yeast *flo8* mutant (YM), yeast *efg1/cph1* mutant (eYM), yeast WT strains (WYM), serum-induced *flo8* mutant (fIM) serum-induced *efg1/cph1* mutant (eIM) and serum-induced WT strains (WIM). ***p < 0.001, by unpaired t test.


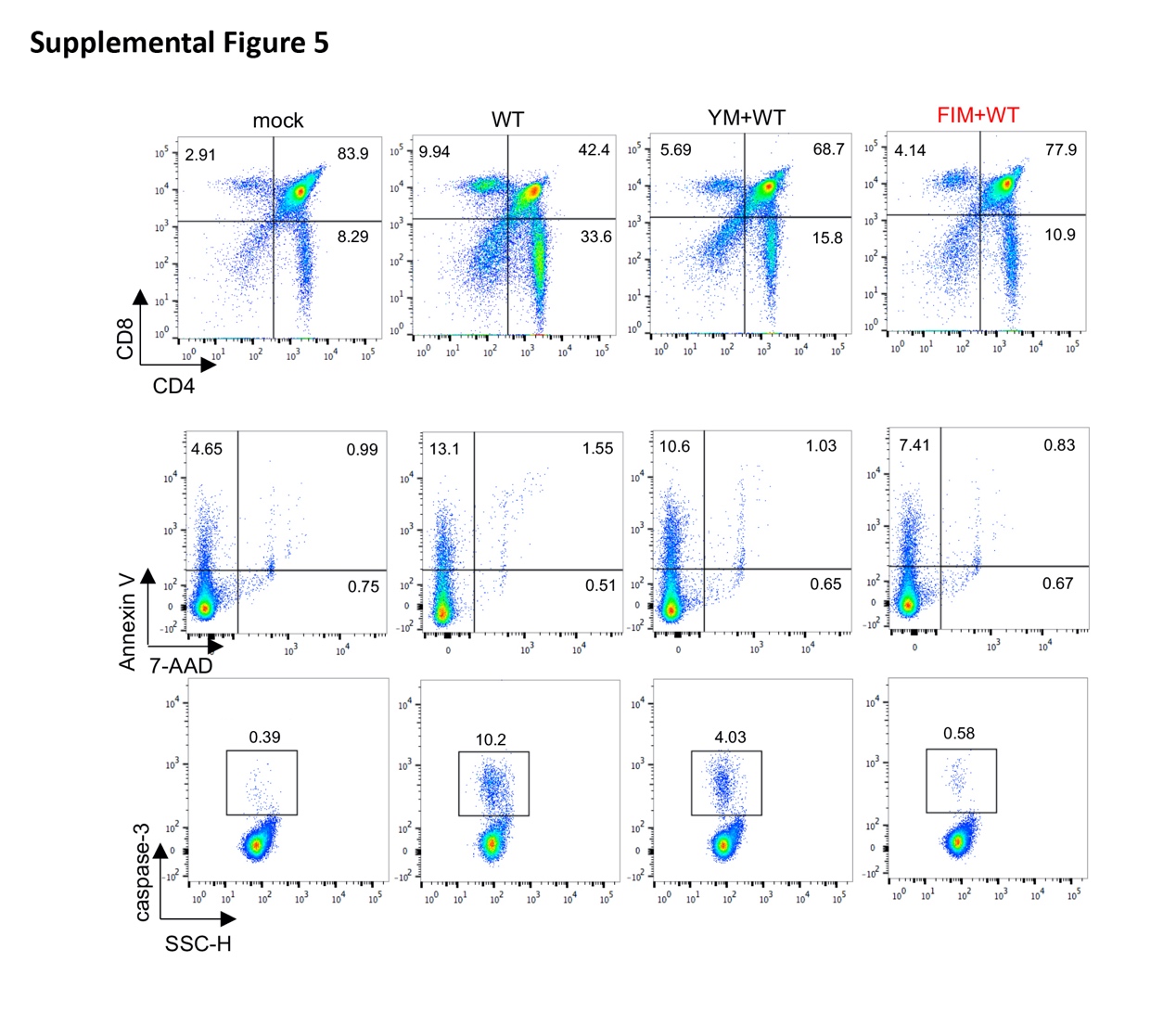


**Fig. S6.** Flow plots represents in Fig. 6D.

**
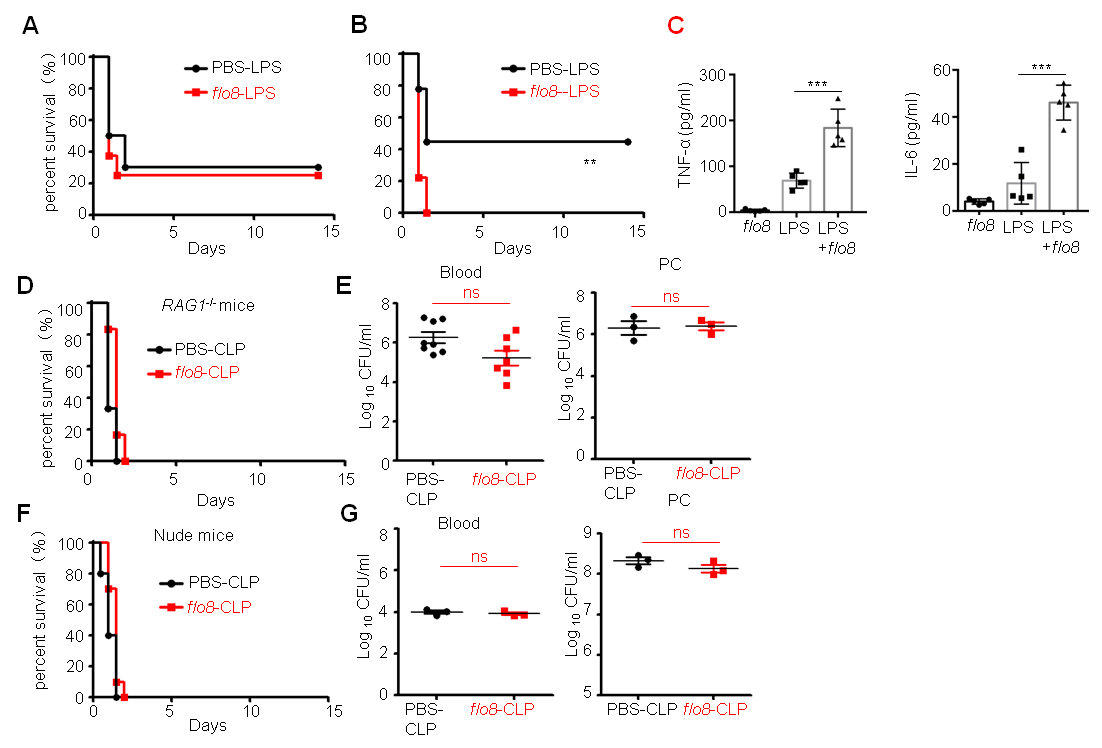
**

**Fig. S7 (A-B)** Survival curves of septic mice caused by LPS treatment. Mice were pre-infected with 5×10^5^ *flo8* mutant for 28 days **(A)** or 7 days **(B)** and injected with 10 mg/kg LPS intraperitoneally (n=8-10). **(C)** ELISA results of TNF-α and IL-6 in serum from mice, which were pre-infected with 5×10^5^ *flo8* mutant for 7 days or not and then injected with 10 mg/kg LPS intraperitoneally for 12 h (n=5). ***p < 0.001, by unpaired t test. **(D-G)** *RAG1*-/- or nude mice were pre-infected with of 5 × 10^5^ CFUs of *flo8* mutant or PBS for 7 days, and then subjected to CLP. Survival curves and microbial burden in the blood or peritoneal cavity (PC) of septic *RAG1*-/- **(D, E)** or nude **(F, G)** were determined. ns, not significant, by unpaired t test.

**Table S1.** Materials used in this study.

| REAGENT or RESOURCE | | | SOURCE | | IDENTIFIER |
| --- | --- | --- | --- | --- | --- |
| Antibody | | |  | |  |
| Fixable viability Dye eFluor 780 | | | eBiocience | | #65086514 |
| Anti-mouse CD45 FITC | | | Biolegend | | #103108 |
| Anti-mouse 3e FITC | | | Biolegend | | #100204 |
| Anti-mouse CD4 V450 | | | eBioscience | | #48004282 |
| Anti-mouse CD8a APC | | | Biolegend | | #100712 |
| Anti-mouse CD11b Percp-cy5.5 | | | BD | | #550993 |
| Anti-mouse Ly6G V450 | | | eBioscience | | #48593182 |
| Anti-mouse CD62L Percp-cy5.5 | | | BD | | #560513 |
| Anti-mouse CD45RA PE | | | BD | | #553380 |
| Anti-mouse IL-10 Percp-cy5.5 | | | Biolegend | | #45710180 |
| Anti-mouse IFN-γ FITC | | | eBioscience | | # 11-7311-82 |
| Anti-mouse IL-17A PE | | | eBioscience | | # 12-7177-81 |
| PE active Caspase-3 Apoptosis Kit | | | BD | | #550914 |
| PE Annexin V Apoptosis Detection Kit I | | | BD | | #559763 |
| Syk Rabbit Ab | | | CST | | #2712BC |
| p-syk(Tyr323) Ab | | | Santacruz | | #B0315 |
| SAPK/JNK Ab | | | CST | | #9252S |
| p-SAPK/JNK(T183/Y185) Rabbit Ab | | | CST | | #4668S |
| PKC delta Rabbit Ab | | | CST | | #2058S |
| p-PKC delta Rabbit Ab | | | CST | | #2055s |
| IκBα Antibody | | | CST | | #9242 |
| Phospho-IKKα/β (Ser176/180) Rabbit mAb | | | CST | | #2697S |
| Ultra-LEAF™ Purified anti-mouse CD210 (IL-10 R) Antibody | | | Biolegend | | #112711 |
| anti-mouse Ly-6G antibody (clone 1A8) | | | Biolegend | | #127649 |
| anti-mouse CD4 antibody (clone GK1.5) | | | Biolegend | | #100442 |
| rat anti-mouse IFN-γ antibody (clone XMG1.2) | | | Biolegend | | #505847 |
| Rabbit IgG | | | Meilunbio | | #MB2301 |
| ELISA | | |  | |  |
| Mouse IL-6 ELISA Ready-SET-Go | | | eBioscience | | # 88-7064-88 |
| Mouse TNF alpha Readey-SET-Go | | | eBioscience | | # 88-7324-88 |
| Mouse IL-10 ELISA Ready-SET-Go | | | eBioscience | | # 88-7105-88 |
| Mouse IFN-γ ELISA Ready-SET-Go | | | eBioscience | | # 88-7314-88 |
| Chemicals, enzymes, Kits | | |  | |  |
| R406 | | | Selleckchem,Houston,USA | | #S2194 |
| SP600125 | | | Selleckchem,Houston,USA | | #S1460 |
| LY240981 | | | Selleckchem,Houston,USA | | #S7697 |
| Ac-DEVD-CHO | | | MCE | | HY-P1001 |
| D-Galactose | | | Sigma | | #G0750 |
| D-Arabinose | | | Sigma | | #141127 |
| L-Rhamnose | | | Sigma | | #R3875 |
| L-Fucose | | | Sigma | | #F2252 |
| D-Mannose | | | Sigma | | #M2069 |
| D-Xylose | | | Sigma | | #X1500 |
| D-Glucose | | | Sigma | | #G7528 |
| Trifluoroacetic acid | | | Sigma | | #302031 |
| 1-Methylimidazole | | | Sigma | | #336092 |
| Recombinant Murine IL-10 | | | Peprotech | | #210-10 |
| Recombinant Murine GM-CSF | | | Peprotech | | #315-03 |
| Concanavalin A, Alexa Fluo 48 Conjugate | | | Thermo Fisher | | #C11252 |
| Collagenase Type 2 | | | Worthington | | #LS004176 |
| PrimeScript™ RT Master Mix | | | Takara | | #RR036A |
| SYBR® Premix Ex Taq™ II | | | Takara | | #RR820A |
| Micro BCA Protein Assay Kit | | | Sangon BIotech | | #C503061 |
| ToxinSensor Chromogenic LAL Endotoxin Assay Kit | | | Genscript | | #L00350C |
| Oligonucleotides | | |  | |  |
| Ddit4-F CAAGGCAAGAGCTGCCATAG  Ddit4-R CCGGTACTTAGCGTCAGGG | | | | GENEWIZ | N/A |
| Bim-F CCACCTCTGCCTCTTAAGT AAC  Bim-R TCCTCCTTTAGGCTCTCCTTAG | | |  | |  |
| p53-F GCGTAAACGCTTCGAGATGTT  p53-R TTTTTATGGCGGGAAGTAGACTG | | |  | |  |
| Fas-F TATCAAGGAGGCCCATTTTGC  Fas-R TGTTTCCACTTCTAAACCATGCT | | |  | |  |
| FasL-F TCCGTGAGTTCACCAACCAAA  FasL-R GGGGGTTCCCTGTTAAATGGG | | |  | |  |
| Bax-F TGAAGACAGGGGCCTTTTTG  Bax-R AATTCGCCGGAGACACTCG | | |  | |  |
| BCLXL-F TTCGGGATGGAGTAAACTGGG  BCLXL-R AGTCATGCCCGTCCACAAAA | | |  | |  |
| Bcl2-F ATGCCTTTGTGGAACTATATGGC  Bcl2-R GGTATGCACCCAGAGTGATGC | | |  | |  |
| GAPDH-F AGGTCGGTGTGAACGGATTTG  GAPDH-R TGTAGACCATGTAGTTGAGGTCA | |  | | |  |
| TREC-F CAAGCTGACAGGGCAGGTTT  TREC-R TGAGCATGGCAAGCAGTACC | |  | | |  |
| Biological Samples |  | | | |  |
| *C.albicans* SC5314 |  | | | | N/A |
| *flo8* | Cao et al 2006 | | | | N/A |
| *flo8/FLO8* | Cao et al 2006 | | | | N/A |
| *efg1*/*cph1* | Chen et al | | | | N/A |
| UCA3 |  | | | | N/A |
| UCA21 |  | | | | N/A |
| Experimental Models |  | | | |  |
| *Card9*^-/-^ mice | Jia et al 2014 | | | | N/A |
| *Clec7a*^-/-^ mice |  | | | |  |
| *Clec4n*^-/-^ mice |  | | | |  |
| *Clec4d*^-/-^ mice |  | | | |  |
| *RAG1*^-/-^ mice |  | | | | N/A |
| C57BL/6 | SHANG HAI SLAC | | | |  |
| Balb/C | SHANG HAI SLAC | | | |  |
| Software and Algorithms |  | | | |  |
| GraphPad Prism 6.0 | Graphpad | | | | N/A |
| Microsoft Excel | Microsoft | | | | N/A |
| FlowJo (v.7.6.1) | FlowJo | | | | N/A |
| MassHunter GC/MS Acquisition Software (B.07.00) | Agilent Technologies | | | | N/A |
|  |  | | | |  |
